# Plant Feed Additives as Natural Alternatives to the Use of Synthetic Antioxidant Vitamins on Livestock Mammals’ Performances, Health, and Oxidative Status: A Review of the Literature in the Last 20 Years

**DOI:** 10.3390/antiox10091461

**Published:** 2021-09-14

**Authors:** Carmen L. Manuelian, Rosario Pitino, Marica Simoni, Alexandros Mavrommatis, Massimo De Marchi, Federico Righi, Eleni Tsiplakou

**Affiliations:** 1 Department of Agronomy, Food, Natural Resources, Animals and Environment, University of Padova, Viale dell’ Università 16, 35020 Legnaro, Italy; carmenloreto.manuelianfuste@unipd.it (C.L.M.); massimo.demarchi@unipd.it (M.D.M.); 2 Department of Veterinary Science, University of Parma, Via del Taglio 10, 43126 Parma, Italy; rosario.pitino@unipr.it (R.P.); marica.simoni@unipr.it (M.S.); 3 Laboratory of Nutritional Physiology and Feeding, Department of Animal Science, School of Animal Biosciences, Agricultural University of Athens, Iera Odos 75, GR-11855 Athens, Greece; mavrommatis@aua.gr

**Keywords:** plant extract, essential oils, plant by-product, natural vitamins, synthetic vitamins, vitamin E, vitamin C, tocopherols, tocopheryl, antioxidants

## Abstract

In the last two decades, the interest in natural plant feed additives (PFA) as alternatives to synthetic vitamins in livestock nutrition has increased. After a systematic review, a total of 19 peer-reviewed papers published between 2000 and 2020 were retained to evaluate the antioxidant effects of PFA compared to synthetic antioxidant vitamins (mainly vitamin E; VitE) in livestock nutrition. These studies demonstrated that PFAs could be as efficient as VitE in counteracting oxidative stress in pigs, rabbits, and ruminants. However, PFAs only positively affected animals’ growth performance and feed efficiency in some monogastric studies. The PFA can affect antioxidant enzyme activity in a dose- and method of administration-dependent manner. The antioxidant capacity of both PFA and VitE were depressed in cows fed with diets rich in polyunsaturated fatty acids. Variability among studies could be related to species differences. Despite the interest of the feed industry sector in PFA, there are still very few studies evaluating their antioxidant effect in species other than poultry.

## 1. Introduction

Feed additive supplements, such as vitamins, play a strategic role in livestock management. In recent decades, a growing interest in natural plant feed additives (PFA) is driving research towards plant extracts, essential oils (EO), and by-products of plant origin as alternatives to synthetic vitamins in livestock nutrition for their potential positive effects on animal health and productivity [1,2,3,4]. In critical areas (e.g., Africa), this application can be advantageous for its low economic impact and for the presence of autochthonous plants which can resist severe weather conditions [5,6].

The goal when formulating animal feeds is to replace synthetic vitamins E (VitE) and C (VitC), which are dietary exogenous antioxidants that improve cellular membrane stability [7]. Several PFAs (e.g., flavonoids, phenolic acids, stilbenoids, epicatechin, hesperidin, and quercetin) have demonstrated antioxidant activity [4]. Flavonoids represent the major polyphenol group and contain several compounds of interest for animal feeding [8]. Flavonoids have the capacity to trap free radicals, including reactive nitrogen species and reactive oxygen species (ROS), and chelating metals, whereas phenolic acids act as antioxidants mainly by scavenging free radicals. The latter activity depends on the location and number of hydroxyl (OH) groups located on the flavonoid skeleton [7]. If more OH groups are present in the flavonoid skeleton, the PFA can potentially exert a stronger antioxidant activity because it can donate more electrons. Another important mode of action of the antioxidant components of PFAs is their ability to donate hydrogens to metals, thus inhibiting their pro-oxidative activity [9]. Other mechanisms by which PFA may exert an antioxidant activity in animal tissues are by quenching O_2_, decreasing O_2_ concentrations, thereby preventing peroxide formation, and by activating antioxidant enzymes [10]. Recently other mechanisms have been investigated to explain the antioxidant activity of PFA, namely interactions with specific proteins of the intracellular signaling cascades, modulation of the expression and activity of key proteins, influence on epigenetic mechanisms, or their effect on the gut microbiota [11]. Essential oils, plant extracts, and plant by-products (e.g., from the grape or citrus industries) can be freely fed to livestock reared under both conventional and organic conditions [11,12,13,14]. Furthermore, PFAs are considered natural, harmless, and residue-free; hence, they could meet both consumer expectations and the need of the feed industry to find valid alternatives to synthetic antioxidants.

In livestock mammals, the impact of PFA on the oxidative status of the animal has been investigated less than in poultry [15]. Indeed, in the poultry sector, PFAs have been widely studied in comparison with synthetic vitamins in order to assess their antioxidant activity on both animals’ performances [16] and products traits [17]. In swine, PFA has been mostly tested as alternatives to antimicrobials rather than as antioxidants, particularly after the ban of in-feed antibiotic use in 2006 [18,19,20], whereas in ruminants, PFA (usually essential oils) have been widely investigated as rumen modifiers [21,22]. Nevertheless, some trials comparing natural to synthetic antioxidants have also been conducted, in swine, rabbits, and ruminants, in order to assess their capacity in ameliorating products yield and quality [23]. However, only a few studies have been conducted in livestock to evaluate the antioxidant role of PFAs in comparison to synthetic molecules in affecting their performances. Nevertheless, it should be highlighted that controversy exists in the literature about the true effectiveness of PFA when compared with VitE, probably because they have different efficacies and modes of action [8]. Thus, it is important to summarize the results of their use in monogastrics and ruminants nutrition. The present review focused on the antioxidant capacity of plant extract, EO, and plant by-products as potential alternatives to synthetic antioxidant vitamins in livestock mammals, including their effect on health, performance, and physiological parameters.

## 2. Literature Selection Criteria

A systematic review of peer-reviewed studies published in Pubmed (www.ncbi.nlm.nih.gov; last accessed on 10 January 2021), ISI Web of Science (www.webofknowledge.com; last accessed on 10 January 2021), and ScienceDirect (www.sciencedirect.com; last accessed on 10 January 2021) databases was performed covering a time-span of 20 years (January 2000 to December 2020). The keywords used for the search were: plant extract, plant by-product, essential oil, natural vitamins, synthetic vitamins, swine, pig, rabbit, ruminant, small ruminant, beef, cattle, cow, sheep, goat, livestock, vitamin E, vitamin C, tocopherols, tocopheryl, antioxidant, natural antioxidant, and organic farming. First, documents were selected based on the title and then on the abstract. If the objective of the work and the tested parameters were in agreement with the selection criteria and consistent with the aim of the present review, the articles were downloaded and summarized in the tables that were then described and discussed in the text. The following selection criteria were established: (i) comparisons of the effects either of plant extract, EO, or plant by-products from different agro-industries, with a specific dose of synthetic antioxidant vitamins -or antioxidants in general- in livestock nutrition and (ii) the use of additives of plant origin (excluding propolis and algae). The final database consisted of a total of 22 papers. Most of the research has been conducted in swine (*n* = 12), followed by ruminants (*n* = 8) and rabbits (*n* = 2). Essential oils and extracts of rosemary and oregano (*n* = 5) and grape by-products (*n* = 2) were the most investigated PFA. In fact, a consistent variability of PFA typology across the papers selected was found. Based on the selected available literature, the present review was divided into two different sections according to the animal species we found (monogastrics, including pigs and rabbits; and ruminants). Then, based on the tested parameters, a further division was created concerning the impact of PFA on feed intake, growth, other productive performance parameters, metabolic parameters, and oxidative status for the two categories of animal species. Since data were also available on the impact of PFA on hematological parameters and stress-related gene expression in pigs and rabbits only, it was decided to include this information together with the metabolic and oxidative status parameters.

## 3. Lipid Peroxidation, Free Radical Generation, and Antioxidant Defense System

Cells produce ROS and reactive nitrogen species as a result of their physiological metabolism or as signaling molecules capable of regulating substantial homeostatic functions [24]. However, their overproduction could induce a cascade of oxidative imbalances [24]. It seems that there are two faces of ROS, redox signaling and oxidative stressor, contributing to both physiological and pathological conditions [25]. Within the range of low to moderate concentration levels, ROS regulate normal cell function, whilst at their highest concentrations, their biochemical instability damages cell components such as lipids, proteins, and DNA, resulting in oxidative stress [26]. The induction of oxidative stress and the over-generation of ROS may be crucial triggers that affect livestock, making animals susceptible to diseases [26]. Stress arises in animals in response to unavoidable or adverse environmental conditions (physical, chemical, biological, and psychological stressors) as well [27]. Physical stressors include fluctuations in ambient temperature as well as mechanical injuries [27]. Notably, high ambient temperature is one of the pivotal factors affecting the productivity and the health of livestock [27]. Heat stress causes a decrease in the concentration of fat-soluble vitamins and increases the concentration ROS through the signaling of heat shock proteins [28], severely compromising the antioxidant/pro-oxidants balance.

The initial product of these oxidative imbalances is considered to be the superoxide anion (O_2_^•−^), a severe reactive radical which is generated either through the mitochondrial respiratory chain or phagocytic nicotinamide adenine dinucleotide phosphate oxidase [29]. However, the organism regulates an efficient network of mechanisms to counteract the detrimental effect of ROS, which includes enzymatic or endogenous and non-enzymatic (exogenous) factors [30]. More specifically, the superoxide anion is neutralized to hydrogen peroxide (H_2_O_2_) through the activity of superoxide dismutase (SOD) at an extremely efficient rate [31]. The formed hydrogen peroxide could be either further neutralized via the antioxidant enzymes catalase (CAT) and glutathione peroxidase (GSH-Px) or form other ROS [26]. Amongst these ROS, the generation of hydroxyl radicals (HO^•^) which are originated as a consequence of the Fenton reaction (H_2_O_2_ + Fe^2+^ → Fe^3+^ + OH^−^ + OH) [32], appears to exert a detrimental effect in cellular homeostasis. More specifically, the hydroxyl and perhydroxyl radicals initiate the lipid peroxidation of the polyunsaturated fatty acids generating deleterious aldehydes, namely malondialdehyde (MDA) and 4-hydroxy-2-nonenal [33]. On the other hand, the high accumulation of hydroxyl radicals could also inhibit the activity of CAT and consequently suppress the neutralization of hydrogen peroxide [34]. Conversely, it has been observed that high concentrations of H_2_O_2_ could inhibit GSH-Px activity as well [35]. Interestingly, further to the crucial impair of lipid peroxidation per se, its metabolites, in combination with the formed ROS, are able to oxidate the side chain of amino acids forming proteins carbonyls [36]. Additionally, it has been reported that proteins carbonyls which are formed through the lipid peroxidation’s aldehydes (such as MDA) are more frequently observed [37].

Considering the above, it is plausible to assume that although cells have an efficient and complex mechanism to neutralize the harmful ROS and their by-products, the rapid biotransformation of these molecules could disturb the organism’s homeostasis. Thus, it is of high importance to prevent the detrimental consequences of oxidative stress in its initial step exploiting a wide collection of natural antioxidants.

## 4. Potential Plant Extracts and Plant By-Products as Alternative Sources of Vitamins in Animal Feeds

### 4.1. Monogastrics

#### 4.1.1. Effects on Feed Intake, Growth, and Other Productive Performance Parameters in Pigs and Rabbits

There is evidence to suggest that herbs, spices, and various plant extracts have appetite- and digestion-stimulating properties and antimicrobial effects [38]. Plant extracts contain different molecules with intrinsic bioactivities on animal physiology and metabolism. Several studies have reported significant effects of PFA on animals’ feed intake, growth, and productive performances (Table 1). Plant extract effects may be due to the greater efficiency in the utilization of feed, which enhances growth.

In finishing pigs, dietary supplementation with oregano oil (OO; at 0.0025% of the diet) for 28 days improved their average daily gain (ADG) (by 10.2%) and reduced (by 8.6%) their feed conversion rate (FCR) compared with the basal diet. Animals supplemented with VitE (200 mg/kg) scored intermediate values between the OO and the unsupplemented pigs. Moreover, final weight gain (WG) tended to be greater (by 2.81%) in the OO than in the control group [39]. When fattening pigs were fed with OO and quercetin (QU, 98% extract from *Sophora japonica* L.), during a 28 days trial, growth parameters were significantly affected [40]. More specifically, greater final body weight (BW) was achieved in pigs fed with OO (at 0.025%) or QU (0.025%) diets compared with those consuming a basal diet containing a low level of VitE (by 4.2% and 3.5%, respectively). A similar improvement was observed in ADG (by 18.6% and by 13.4%, respectively). Although average daily feed intake (ADFI) was not affected by feeding experimental diets, Feed:Gain (F:G) ratio was higher in the OO group compared with the control (13.8%) and VitE (12.3%) groups (Table 1) [40].

Dietary supplementation with oregano extract (OE, at 2.0% of the diet) or oregano (*Origanum vulgare*) and rosemary extract (*Rosmarinus officinalis*) (both at a concentration of 0.1%) for 50 days improved the BW of rabbits (by an average of 2.87%) and their ADG (by 5.1%) compared with those consuming a basal diet that included 50 mg/kg of VitE [41]. The results suggest that supplementation with OO can be more effective than VitE in improving growth and some productive performance parameters. The main components of OO are thymol and carvacrol (polyphenols), with their precursors ρ-cymene and γ-terpinene being the most abundant monoterpenes. These results could be explained by the capacity of polyphenols to exert antioxidant effects within the gastrointestinal tract, where they come into direct contact with the cells without being absorbed and metabolized [42], with possible protective action on the gut mucosal cell membranes. Studies comparing the antioxidant properties of Mediterranean food spices and common food additives have shown that extracts from oregano were more effective than butylated hydroxyanisole and butylated hydroxytoluene in inhibiting lipid peroxidation [43]. In vivo, thymol shows the ability to protect the microvilli, which are responsible for the absorption of nutrients [43]. Moreover, unabsorbed polyphenols flow to the hindgut, where they can modulate the gut microbial community acting as prebiotics [44]. Antibacterial effects have, in fact, been reported for thymol and carvacrol from oregano, for example, against *Clostridium perfringens*, *Pseudomonas aeruginosa*, and *Staphylococcus aureus* [45]. Moreover, the higher average daily gain (ADG) and greater feed conversion rate achieved in the studies described could be attributed to the positive effects of extracts on nutrient digestibility. Some authors speculated that herbs could develop their initial activity by adding flavor and, therefore, influencing the eating pattern, total feed intake, and secretion of digestive fluids [43,46].

In addition, ADG was greater (by 4.3%) in green tea catechins than in the control groups (fed a basal diet including grass meal at 10% or 20% in the weaning and finishing phase, respectively) [30]. In fact, among several flavonoids (rutin, dihydroquercetin, quercetin, epigallocatechin gallate, and epicatechin gallate), catechins showed the greatest capacity to inhibit microsomal lipid peroxidation [7]. The oxidation processes can, in fact, decrease the palatability and the nutritional value of feeds and the use of antioxidant vitamins (i.e., VitE) is considered the first defensive line against lipid peroxidation in tissues, protecting cell membranes from free radicals damage which ultimately influence ADG especially during the starter period [47]. Similar to vitamins, the flavonoids tested are able to chelate Fe^2+^, Fe^3+^, and Cu^2+^ and are effective O_2_^•−^ scavengers to varying degrees [7] which can explain their impact on ADG.

In contrast to these findings, the supplementation of diets with OO (at 0.01% or 0.02%) for 42 days did not significantly affect neither the growth nor the feed efficiency parameters in rabbits compared with those receiving a basal (only vitamin premix) or a VitE enriched diet (200 mg/kg) [48]. In young growing pigs, no differences in feed intake (FI) and BW were observed after supplementing the diet for 14 days with a propylene glycol extract of Marigold (*Calendula officinalis* L.; at 3 mL/day) or with a plant extract mixture (containing: carvacrol 5%, cinnamaldehyde 3%, and capsicum oleoresin 2% wt/wt, at 271.2 mg/kg) compared with those supplemented with VitE (100 mg/kg or 90.4 mg/kg) [35,36]. No differences among the ADG, final BW, and FI values were reported when pig diets were supplemented with rosemary extract (RE; at 0.04%), alone or in combination with gallic acid (at 0.02%) or VitE (at 0.04%) [49]. Weaned pigs supplemented with green tea catechins (at 0.2%) did not vary their feed consumption (FC) compared with pigs fed four control diets and with VitE (200 mg/kg). No significant differences were found in pigs’ ADG, FI, and feed:gain (F:G) ratio when fed a basal diet enriched with sunflower oil alone, or linseed oil alone, or linseed oil with olive leaves (OL; at 0.5 or 0.10%) or linseed oil with VitE (200 mg/kg) simultaneously for 90 days [50]. In another study, the dietary supplementation with OL extract (OLE1: 0.000384%; OLE2: 0.00384%; OLE3: 30.096%) did not change the final BW of piglets or their WG compared with two control diets containing the same level of VitE but different fat contents (one had a high fat content due to the inclusion of 8.3% of linseed oil) [51]. Nevertheless, ADFI was lower in OLE1 (by 10.5%), OLE2 (by 10.8%), and OLE3 (by 10.5%) in comparison with the low fat diet fed group. However, the OLE3 group had lower F:G ratio (by 11.0%) compared with the control group [51] (Table 1).

The inconsistency of some of the results described above can be explained by the generally low and variable bioavailability of PFA. Their absorption at the stomach level is limited due to their glycosylated form because most of them need to be hydrolyzed in the intestine by the microbial population or by enzymes in order to be absorbed; furthermore, they can be extensively metabolized at the hepatic level [42]. Moreover, although aromatic herbs and essential oils can improve taste and palatability, thereby increasing consumption, in some cases, the opposite was found. Some authors have, in fact, also shown that as the concentration of dietary OEO increases, consumption decreases [52].

**Table 1:** Effects of plant feed additives on feed utilization and growth parameters in monogastrics.

PFA	PFA Tested Dose (% in the Diet)	Animal Species Involved	Vitamin/Pro-Vitamin Compared	Type of Parameter	Trait Evaluated	Comparison to Negative Control: Effect (PFA Dose)	Comparison to Positive Control: Effect (PFA Dose)	Period of Study (Days)	Reference
Oregano essential oil	0.025 + 200 mg/kg VitE	Pigs	E, 200 mg/kg	GROPerf	IBW, FBW,	NS	NS	28	[39]
					ADG	↑ 10.2%	NS		
				FUPerf	F:G	8.6%	NS		
					ADFI	NS	NS		
Oregano plant (OP) and Rosemary (RP) aqueous extracts	0.2 (OP), 0.2 (RP), 0.1 OP+ 0.1 RP(OPRP)	Rabbits	E, 150 mg/kg	GROPerf	BW(30 d)	NS	NS	50	[41]
					BW (80d)	↑ 2.94% (OP) ↑ 4% (OPRP)	↑ 2.1% (OP) ↑ 3.14% (OPRP)		
					ADG	↑ 5.14% (OP) ↑ 5.14% (OPRP)	↑ 3.37% (OP) ↑ 3.37% (OPRP)		
				FUPerf	FI	NS	NS		
					F:G	NS	NS		
Oregano essential oil (OEO)	0.1, 0.2	Rabbits	E, 200 mg/kg	GROPerf	IBW, FBW, WG	NS	NS	42	[48]
				FUPerf	FI, F:G	NS	NS		
Rosemary extract (RE)	0.04 (RE), or + 0.02 gallic acid (REG) or + 60 mg/kg of VitE (REE)	Pigs	E, 60 mg/kg	GROPerf	ADG, FBW	NS	NS	115, 122	[49]
				FUPerf	FI	NS	NS		
Olive leaves	0.5, 1	Pigs	E, 200 mg/kg	GROPerf	ADG	NS	NS	90	[50]
			(2 control diet, 1 with sunflower oil, 1 with linseed oil)						
				FUPerf	FI, F:G	NS	NS		
Olive leaf extract	0.000384 (OLE1),0.00384 (OLE2), 0.096 (OLE3)	Pigs	E, 105 IU/day	GROPerf	IBW, FBW, WG	NS	NS	21	[51]
			2 control diet, 1 low fat, 1 enriched with linseed oil						
					ADFI	Control low fat: ↓ 10.47% (OLE1) ↓ 10.84% (OLE2) ↓ 10.47% (OLE3)	NS		
					F:G	Control low f: ↓ 10.97% (OLE3)			
OEO, quercetin (Q)	0.025		E (200 mg/kg)	GROPerf	IBW	NS	NS	28	[40]
					FBW	↑ 3.54% (Q) ↑ 4.25% (OEO)	NS		
					ADG	↑ 13.4% (Q) ↑ 18.56% (OEO)	NS		
					Shrinkage, % of BW	↓ 35.98% (OEO)	NS		
				FUPerf	ADFI	NS	NS		
					F:G	↓ 13.75% (OEO)	↓ 12.32% (OEO)		
Grape seed polyphenols	0.02, 0.03	Swine	E (200 IU/kg)	Reproductive performance of sows	D of pregnancy, Total born, N born alive, N mummies, Total live piglet BIW, Average live piglet BIW, Average weight of weaned piglet, Average weight gain of piglet, N weaned piglet by sow, Weaning weight by sow	NS	NS	56	[53]
Green tea catechins	0.02	Pigs	E 200 mg/kg	FUPerf	FC		NS	129	[54]
			4 control groups: 1 without grass meal (C), 3 with different % of GM (GM105, GM50, GM180)						
				GROPerf	ADG	GM105: ↑ 4.3%	NS		

Abbreviations: ADFI: average daily feed intake; ADG: average daily gain; BW (n.): body weight (interval); F:G: feed to gain ratio (see also FCR); FBW: final body weight; FI: feed intake; FUPerf: feed utilization performances; GROPErf: growth performances; IBW: initial body weight.

#### 4.1.2. Effects on Metabolic and Hematological Parameters, Oxidative Status, and Gene Expression in Pigs and Rabbits

Several PFAs have been shown to affect metabolic and hematological parameters, as well as health, fertility, and oxidative status in monogastrics (Table 2). In pigs, transport stress has been demonstrated to increase the levels of cortisol and creatine kinase in serum [39]. However, the dietary supplementation of finishing pigs with OO (at 0.0025%) mitigated this effect, reducing serum cortisol and creatine kinase in comparison with the control group (no supplementation, no stress). Dietary supplementation with VitE (200 mg/kg) resulted in intermediate values of serum cortisol and reduced creatine kinase compared with the stressed group but did not reach the same level as the control group [39]. Serum cortisol is a known marker of stress and creatine kinase of muscle damage. Increases in metabolic rate as part of the acute stress response may result in higher oxidative damage [55]. The oxidative damage can be efficiently mitigated by OO polyphenols, whose antioxidant properties are well known. They are able, in fact, to chelate pro-oxidant metal ions and to donate hydrogens in relation to their –OH groups number and arrangement on the ring(s) but also to their lipid/hydrophilic phase partitioning [7]. Muscle heat shock protein (HSP) 27 and the mRNA expression levels of the *HSP 70* gene in the jejunum muscle were also increased after 5 h of transportation stress [39]. Pigs that were supplemented with OO revealed a similar expression level to that of control animals (neither transported nor supplemented). On the other hand, the dietary supplementation with VitE was not effective in reducing the mRNA expression levels of *HSP 27* and *HSP 70* genes induced by transportation stress [39]. The HSP are proteins that protect cells from stress preventing protein aggregation, restoring the function of damaged proteins, and inhibiting denaturation [56], and the cellular damage derived from ROS accumulation is considered as a key factor in the activation of HSP genes [57]. These results indicate that the dietary supplementation with OO in comparison with VitE is more effective in decreasing the stress response induced by transportation as a function of their active compounds free radical-scavenging ability [7]. Young growing pigs that were fed a high- polyunsaturated fatty acids (PUFA) diet presented oxidative DNA damage in peripheral blood lymphocytes (measured with the comet assay) and increased plasma and 24 h urinary malondialdehyde (MDA) content, which indicates lipid peroxidation [58,59]. Dietary supplementation with Marigold extracts whole flower tops or petals (at 3 mL/day) [58], which is one of the best commercial sources of pure lutein and zeaxanthin [60], or a plant extract mixture (carvacrol, cinnamaldehyde, and capsicum oleoresin; at 0.027% of the diet) [59] for 14 days reduced the DNA percentage in the tail of the comet and olive tail moment (OTM)—indicators of DNA oxidation damage—similarly to VitE. The decrease induced by Marigold extract from petals was similar in magnitude to the one observed for the plant extracts mixture being around 45% for the DNA percentage in the tail of the comet and around 70% for OTM (Table 1). However, only the Marigold extract from petals and the plant extract was able to reduce the urinary 8-hydroxy-2¢-deoxyguanosine (8-OHdG) excretion compared with the group without supplementation (by an average of 45.6%), while VitE was not effective (Table 1). The dietary supplementation with Marigold extract, plant extract, or VitE could not prevent the increase of plasma and 24-h MDA values when pigs were fed a high-PUFA diet [58,59]. Comparing both studies, the same trend and similar values were observed for the 24-h urinary iPF2α-VI (urine isoprostanes) among the low-PUFA diet, high-PUFA diet, Marigold extract from petals or plant extract mixture, and VitE enriched ones. Although the 24-h urinary iPF2α-VI did not statistically differ among the feeding groups when evaluating the Marigold extract from petals enriched diet compared with the low-PUFA control one, a numerical decrease in the 24-h urinary iPF2α-VI was found. These results indicate that Marigold extract from petal and plant extracts containing carvacrol, cinnamaldehyde, and capsicum oleoresin was as effective as VitE in preventing DNA oxidation. Treated lens epithelial cells with lutein or zeaxanthin can effectively block H_2_O_2_ induced protein oxidation, lipid peroxidation, and DNA damage as demonstrated in vitro [61]. In addition, dietary supplementation of albino rats with the same molecules led to a significant elevation of anti-oxidant enzyme levels in dose-dependent manner, concomitant with a similar increment of the total anti-oxidant capacity [60]. Moreover, the few differences between the two Marigold extracts used revealed that an extract from petals was more effective in protecting DNA than the extract from flower tops. On the other side, cinnamaldehyde is a phenylpropanoid that demonstrated considerable metal ion chelating ability, which is an important mechanism of antioxidant action, and lower to moderate free radical scavenging activity [62].

A diet enriched with linseed oil, which induced a state of n-3 PUFA oxidative stress in young pigs, was supplemented over a period of 14 days with sweet chestnut wood extract (*Castanea sativa* Mill., SCW; at 0.075%, 0.15%, and 0.3% of the diet) [63]. Chestnut wood extract (CWE) is a source of hydrolyzable tannic acid, a naturally occurring plant polyphenol, composed of a central glucose molecule derivatized at its hydroxyl groups with one or more galloyl residues. Its capacity to inhibit lipid peroxidation has been demonstrated to be comparable to those of butylated hydroxyanisole, butylated hydroxytoluene, α-tocopherol, and trolox in vitro [64]. Despite the antioxidant supplementation, pigs fed the high PUFA diet in the SCW groups, along with the VitE one, had a higher (by 80.5% on average) urinary MDA content compared with those on a low PUFA control diet, while the same parameter was lower (by 31.7%) only in the high SCW group compared with the high PUFA control group. Moreover, feeding pigs with SCW led to an increase (by 32.5%) in plasma MDA content compared with the low PUFA basal diet, whereas the VitE group did not show any significant difference when compared to the same diet. These results show that SCW at low dosages is not able to mitigate the oxidative stress induced by feeding a high PUFA diet in piglets, whereas higher doses of SCW could be successful in reducing oxidation. However, the 24-h urinary iPF2α-VI, TAS, and GSH-Px activity were not significantly affected in this trial. Indexes of DNA damage were also investigated. The percentage of DNA in the tail was similarly decreased in the SCW (by a mean proportion of 29.4%) and VitE (by 45.0%) groups compared with the high PUFA control one. A similar, lower OTM was found for the SCW (by 54.3%) and VitE (by 65.1%) groups. With reference to serum enzymes, pigs fed with SCW exhibited lower alanine aminotransferase (ALT) values (by 32.3% on average) (Table 1). However, no differences in comparison to VitE supplemented group was found [63]. These results confirm the potential of SCW supplements at higher doses to mitigate PUFA oxidation.

Olive leaves are an important source of antioxidants, such as phenolic compounds and flavonoids, which have been demonstrated to inhibits the action of reactive species that participate in cellular biochemical processes and protect human erythrocytes against oxidative damage. These plant compounds are, therefore, effective antioxidants in biological systems, suggesting that their intake may be related to the prevention of oxidative stress in vivo, with consequent health benefits [65]. However, in a 21-day piglet trial, the effect of supplemental OLE in high PUFA diets (OLE1: at 0.000384%, OLE2: at 0.00384%, and OLE3: at 0.096%) on antioxidant status was compared with two control diets (low or high in PUFA content), both deficient in VitE, or with a high PUFA diet supplemented with VitE (105 mg/kg) [51]. Serum oxidized low-density lipoproteins and urinary MDA content were not significantly affected by OLE supplementation. On the contrary, lower values (by an average of 35.3%) of 24 h-urinary iPF2α-VI were found in the VitE fed animals. Piglets supplemented with OLE had a higher urinary MDA value compared with those consuming the low PUFA control (by 45.3%) diet. Pigs fed with the lower level of OLE, compared with the VitE diet, showed higher (by 30.1%) urinary MDA content. Markers of DNA damage were also influenced by dietary treatments. Significantly lower tail DNA percentages (by an average of 16.4%) and OTM (by an average 19.2%) were found in the OLE group compared with animals fed VitE. The urinary 8-OHdG was lower in OLE3 compared with OLE1 (by 31.6%), VitE (by 39.3%), and PUFA control (by 43.5%) diets. Liver enzyme activities [aspartate amino transferase (AST) and ALT] were generally not influenced by experimental diets; only gamma-glutamyl transferase (GGT) was reduced (by 26.6%) in OLE1 compared with high PUFA control fed animals. These results suggest that the dietary inclusion of OLE in piglets at the tested does not exert positive effects on oxidative status.

Dietary supplementation with OO or QU (at 0.025% of the diet) for 28 days significantly improved pig serum, liver, and antioxidant muscle status [40]. The active compounds and properties of OO are described above, and similarly, quercetin is a flavonoid that can scavenge free radicals and chelate metals as well [7,66]. Consistently with this description, ROS were lower in the QU (by 22.9%) and OO groups (by 31.3%) than in the control group and were also lower when compared with the VitE group (by 20.3% on average) (Table 1). Similar trends were found in the liver and in muscle (but no difference with VitE). The TBARS values were also significantly lower in QU and OO groups but only compared to the control group. In another trial, liver TBARS values were also lower in QU (by 32.9%) and in OO (by 25.3%) fed fattening pigs. Furthermore, the SOD activity was higher in QU and OO groups than in controls (by 13.1 and 16.3%, respectively), whereas, in liver and muscle, no significant differences were observed. The dietary inclusion of QU and OO increased the GSH-Px activity in the liver and serum equally. The greatest increase (by 31.0%) was found in the serum of the OO group compared with the control group. Lower serum GSH-Px activity was found in the QU compared with the OO group. No differences in both liver and muscle GSH-Px activity between OO and QU-fed animals were found [40]. Accordingly, in rats, quercetin and carvacrol showed antioxidant properties by increasing hepatic expression and activity of GSH-Px [67,68]. In another study, sows supplemented with grape polyphenols (GPP; at 0.02% and 0.03% of the diet) for 56 days improved their SOD activity in plasma, compared with both the control (by 70.6% on average) and the VitE group (by an average of 52.1%) [53]. The GPP fed animals also ameliorated their GSH-Px activity (by 41.5% on average), but only compared with those consuming the basal diet. In the same study, no significant effects were observed in newborn piglets with regard to some specific traits [53].

From the studies described above, it appears that the effect of PFA on monogastric animals can be influenced by environmental and dietary factors, but also by the dose rate. The latter may affect the PFA metabolism site. It has been shown that polyphenols administered at high doses are metabolized in the liver, while at lower doses, their metabolism takes place in the intestinal mucosa [69]. It is still unclear where PFA and their extracts have direct antioxidant effects in vivo [8], even if some of their mode of action has been partially described. When studying the impact of PFAs on the different organs and tissues -and therefore the potential antioxidant activity exerted at the cellular level-, it should be understood that their concentration in target tissues largely depends on how much they are biotransformed in the liver and small intestine and also on their lipophilicity. In order to mitigate oxidative stress, PFA extracts need to penetrate the lipid membrane, enter the cell, and act at an intracellular level [44]. This process should be accurately studied and quantified in order to optimize their use in animal diets.

### 4.2. Ruminants

#### 4.2.1. Effects on Feed Intake, Growth, and Other Productive Performance Parameters in Ruminants

Little research has been done comparing the effects of PFA and synthetic vitamins on growth, feed efficiency, and performance in ruminants (Table 3). In lambs, dietary supplementation with VitE (225 IU of DL-α tocopherol) once per week or an ethanol extract of saffron (*Crocus sativus* L.) petal extract given by either a subcutaneous injection (25 mg/kg BW) or orally (500 mg/kg BW) in a liquid form at the same frequency showed no significant effect on growth performance, dry matter intake (DMI) or F:G ratio [70]. Neither the addition of grape pomace (at 5.17% and 10.3% of diet DM) or of VitE (at 0.045% of the DM) in lambs’ basal diet, where VitE was already included, affected the growth rates of the lambs [71]. The addition of yerba mate, either alone (at 3%) or in association with VitE (375 IU/kg DM), had no effect on the DMI of lactating cows fed an unsaturated fatty acid-enriched diet in comparison with the basal diet (containing VitE at 375 IU/kg DM) [72]. The administration of *Andrographis paniculata* or turmeric acid (both at the doses of 0.5% DM) did not affect ADFI when compared with both the control and the supplemented group (400 mg/kg DM of VitE). Moreover, these treatments induced a reduction in F:G ratio in comparison with both the control and VitE groups (on average by 19.52% and 19.15% for *Andrographis paniculata* and Turmeric acid, respectively) (Table 1). The feed efficiency increased significantly (by 15.17%) in the *Andrographis paniculata* group, in comparison with the control, while the growth performance was not affected [73]. It should be highlighted here that the leaves of *Andrographis paniculata* contain many bioactive compounds, including diterpene lactones, diterpene glucoside, and flavonoids [74]. On the other side, curcuminoids (curcumin and its related compounds) are a major chemical constituent in turmeric acid, and studies suggest that their activity depends significantly on the introduction of electron-donating groups (methoxy) in the ortho position of the 4-hydroxyphenyl group [75]. This antioxidant action can improve growth performances and feed efficiency, as previously described.

#### 4.2.2. Effects on Metabolic Parameters and Oxidative Status in Ruminants

Some effects of PFA on metabolic and haematological parameters and on antioxidant status have been found in ruminants (Table 4). The dietary supplementation with VitE (225 IU of DL-α tocopherol acetate once per week) or ethanolic saffron petal extract—known for its content of two antioxidant compounds from different chemical families: the carotenoid crocins and the flavonol kaempferol [76] through a subcutaneous injection (25 mg/kg BW) or oral administration (500 mg/kg BW in a liquid form once per week) decreased the lipid oxidation in lambs kidneys significantly as indicated by the MDA values [70]. The subcutaneous injection decreased MDA values by 30.8%, and oral administration reduced the values by 33.5%. This is consistent with their antioxidant action: carotenoids are, in fact, able to inhibit Fe^3+^/AA induced oxidation, scavenge free radicals, and act as reductants, while the flavonol kaempferol has been found to exert a moderately high free radical scavenging action [7]. The subcutaneous injection of ethanolic saffron petal extract appeared to significantly increase the GSH-Px activity (by 45.9% on average) in blood in comparison with the control and the VitE treated group. This is in agreement with recent studies that evaluated the effects of crocin, crocetin, and safranal on oxidative stress, demonstrating a reduction in lipid peroxidation (MDA levels) and the increase in the levels of glutathione, and antioxidant enzymes, including GSH-Px in the animal organism [77]. Moreover, a significant reduction in the blood cholesterol levels of lambs receiving either the ethanolic saffron petal extract by injection—an effect already described in the literature [78] or the VitE supplement was observed in comparison with the control group and those fed the ethanolic saffron petal extract orally, indicating that the way of administration of nutrient supplements might play a significant role in their effectiveness. This hypothesis is further supported by the significantly higher MDA content (1.87 vs. 1.42 nmol/dL) in the blood plasma of the lambs given the ethanol saffron petal extract orally in comparison with the injected ones [70]. In fattening lambs, the dietary supplementation with either VitE (6 g/kg DM) or naringin (1.5 or 3 g/kg DM) resulted in a significant reduction in the TBARS values (by 38.1%, 29.4%, and by 45.1%, respectively) in their blood plasma [79]. Moreover, both VitE and naringin reduced (by 25% to 30%) the serum concentrations of triglycerides in fattening lambs when fish oil was incorporated into their diets for 21 days [79]. Similar decreases of plasma lipid MDA (20–40%) were found after the administration of *Andrographis paniculata* (0.5% DM), turmeric acid (0.5% DM), and VitE (400 mg/kg DM) over four weeks [73]. A similar reduction (by 55%) was also observed after 14 weeks following the administration of *Andrographis paniculata.* Plants rich in polyphenols such as rosemary, grape, grapefruit, and marigold decreased lipid peroxidation in bovine tissues [48], did not affect lipid metabolic parameters (triglycerides, NEFA, and phospholipids) and the activity of the antioxidant enzymes in the liver but caused an increase in the content of plasma conjugated dienes, an effect which needs further investigation [58].

## 5. Final and General Remarks

This review focused on the antioxidant capacity of plant extracts, essential oils, and by-products of plant origin as potential alternatives to synthetic antioxidant vitamins in livestock mammals’ feedstuffs. Natural antioxidants, in comparison to synthetic ones, have been described to show several advantages like having a wide range of solubility and of antioxidant activity and being completely metabolized by the organism. Because of this, they are perceived as innocuous substances, and the interest in their use is increasing even if limited by their high cost. On the other side, synthetic antioxidants are generally inexpensive and widely applied in livestock nutrition; they possess low water solubility and medium to high antioxidant activity, but some of them are stored in the adipose tissue, so safety concerns are rising, and the use of some of them is nowadays banned. VitE is the only synthetic antioxidant that has been compared with PFA in livestock mammals’ trials, and only a few studies in both monogastric and ruminants deal with this issue. Most studies have tested PFA for its effects on feed intake, growth, slaughter, and milk productive performance parameters. A small number of studies have evaluated the impact of PFA on metabolic and hematological parameters, oxidative status, and HSP gene expression. Both positive and negative effects of PFA on several animal functions were found. Oregano essential oil, alone or in association with quercetin, compared with a basal diet, decreased F:G ratio by increasing ADG and final BW in pigs. Moreover, in rabbits, the oregano plant alone or in combination with aqueous rosemary extracts showed positive effects on the above-mentioned parameters in comparison with both basal and VitE rich diets. Marigold (*Calendula officinalis*), plant extract mixture (carvacrol, capsicum oleoresin, and cinnamaldehyde), and oregano essential oil alone or in combination with quercetin, sweet chestnut, or plant extract (grape skin and oregano), respectively, showed a similar action to VitE in reducing oxidative stress with a trend for higher antioxidant enzymes activity in monogastric animals. The PFA did not affect ruminants’ growth performance and feed efficiency. Saffron petal extracts, naringin, *Andrographis paniculata*, turmeric acid, and plants rich in polyphenols in general (rosemary, grape, grapefruit, and marigold) showed similar actions to VitE in reducing lipid peroxidation and, consequently, oxidative stress without affecting biochemical parameters in bovine, ovine, and caprine.

In some cases, the effect seems to be dose-dependent, such as in the case of the grape seed polyphenol extract that induced a linear increase of SOD and GSH-Px activities with increasing inclusion rate in pigs’ diets. The beneficial effects of both PFA and VitE in PUFA rich diets were in general decreased, probably due to the higher susceptibility of the nutrients to oxidation.

Some variability in the results can also be attributed to the animal species (monogastric vs. ruminants). The rumen may mitigate the impact of PFA on the digestion process through the fermentation activity on the dietary substrates with partial modification of the molecules reaching the gut. Considering the presence of these fundamental variables and the enormous number of molecules and formats of PFA available on the market, it should be highlighted here the need for standardization of the evaluation systems that could allow an optimization of the studies in this field, leading to a more efficient and conclusive validation process for these products.

## 6. Conclusions

The PFA and VitE have a similar effect in mitigating oxidative stress in both monogastrics (i.e., pigs and rabbits) and ruminants under moderate stress conditions, which are frequent in intensive livestock farming. The influence of PFA on antioxidant enzyme activities can be dose- or method of administration-dependent. In monogastrics, certain PFAs can have a positive effect on growth performance and, consequently, on feed efficiency. In cows, PUFA rich diets reduced the antioxidant action of both PFA and VitE. Some variability in the results of the studies included in the present review could be related to the differences between animal species but also to the variability of the experimental protocols and of the parameters tested on the animals.

## Figures and Tables

**Table 2:** Effects of plant feed additives on metabolic and hematological parameters, health and fertility, and on oxidative status in monogastrics.

PFA	PFA Tested Dose (% in the Diet)	Animal Species Involved	Vitamin/Pro-Vitamin Compared	Type of Parameter	Trait Evaluated	Comparison to Negative Control: Effect (PFA Dose)	Comparison to Positive Control: Effect (PFA Dose)	Period of Study (Days)	Reference
Marigold (*Calendula officinalis*)	3 mL/day of C. officinalis propylene glycol extracts: 1 from petals (C1) and 1 from whole flower tops (C2)	Young growning pigs	E, 100 mg/kg	Lipid peroxidation	% of DNA in the tail of the comet	Oil diet: ↓ 43.3% (C1) ↓ 31.7% (C2)	NS	14	[58]
					OTM	Oil diet: ↓ 68.8% (C1) ↓ 56.2% (C2)	NS		
					8-OHdG	Oil diet: ↓ 48.4% (C1)	↓ 35.84% (C1)		
					TAS, GSH-Px, Urinary iPF2a-VI	NS	NS		
					Urinary MDA	Control diet: ↑ 113.88% (C1) ↑ 129.22% (C2)	NS		
					Plasma MDA	Control diet: ↑ 36.51% (C2)	NS		
Plant extract, mixture composed of carvacrol, capsicum oleoresin, and cinnamaldehyde	0.02712	Young pigs (Sus scrofa)	E, 90.4 mg/kg	Lipid peroxidation	Urinary MDA	C:↑ 138.1%	NS	14	[59]
					Plasma MDA	C:↑ 29.03%	NS		
					TAS, GSH-Px	NS	NS		
					Urinary iPF2a-VI	↓ 40.52%	NS		
				Lymphocyte DNA damage	Percentage of DNA in the tail of the comet	Oil diet: ↓ 45.83%	NS		
						Oil diet: ↓ 70.21%	NS		
					8-OHdG	Oil diet: ↓ 42.69%	NS		
Olive leaf extract	0.000384 OLE1,0.00384 OLE2, 0.096 OLE3 *	Pigs	E	AA status	Urinary F2-isoprostanes (Urinary iPF2-III); Serum oxLDL (Oxidized low-density lipoprotein); Urinary MDA	NS	NS	21	[51]
					Plasma MDA	Control low f: ↑ 46.48% (OLE1) ↑ 44.97% (OLE2) ↑ 44.13% (OLE3)	↑ 30.3% (OLE1); = OLE2 = OLE3		
				Other AA markers	Plasma	NS	↓ 64.6% (OLE1) ↓ 65% (OLE2) ↓ 69.25% (OLE3)		
					a-tocopherol				
					Plasma γ tocopherol	Control low f: ↑ 447.62% (OLE1) ↑ 391.7% (OLE2) ↑ 311.9% (OLE3)	↑ 120.1% (OLE1) ↑ 97.61% (OLE2)		
					Serum ACW, ACL, Whole blood GSH-Px, Whole blood SOD	NS	NS		
				DNA damage markers	Tail DNA	NS	↓ 18.77% (OLE1) ↓ 14.05% (OLE3)		
					OTM1	NS	↓ 26.07% (OLE1) ↓ 16.84% (OLE2) ↓ 14.6% (OLE3)		
					Urinary 8-OHdG	Control low F: ↓ 43.3% (OLE2) ↓ 43.54% (OLE3)	↓ 39.1% (OLE2) ↓ 39.33% (OLE3)		
				Liver enzymes	Serum AST, ALT	NS	NS		
					Serum GGT	Control high F: ↓ 26.59% (OLE1)	NS		
Oregano essential oil (OEO)	0.02 + 200 mg/kg of VitE	Pigs	E	Biochemical parameters	Serum Cortisol	Control: NS; Transport stressed (TRS): ↓ *p* < 0.05	NS	28	[39]
					Serum creatine kinase (CK)	Control: NS; Transport stressed (TRS): ↓ *p* < 0.05	NS		
Oregano essential oil (OEO), quercetin (Q)	0.025	Pigs	E	AA status serum	ROS (RLU)	↓ 22.94% (Q) ↓ 31.34% (OEO)	↓ 20.27% (OEO)	28	[40]
					TBARS	↓ 26.63% (Q) ↓ 14.75% (OEO)	NS		
					T-SOD	↑ 13.12% (Q) ↑ 16.31% (OEO)	NS		
					GSH-Px	↑ 15.83% (Q) ↑ 31.03% (OEO)	↑ 14.53% (Q) ↑ 29.57% (OEO)		
				AA status liver	ROS	↓ 33.9% (Q) ↓ 25.44% (OEO)	↓ 24.2% (Q)		
					TBARS	↓ 32.91% (Q) ↓ 25.32% (OEO)	NS		
					T-SOD	NS	NS		
					GSH-Px	↑ 14.62% (Q) ↑ 13.34% (OEO)			
				AA status muscle	ROS	↓ 20.96% (Q) ↓ 17.88% (OEO)	NS		
					TBARs	↓ 20.39% (Q) ↓ 19.42% (OEO)	NS		
					T-SOD, GSH-Px	NS	NS		
Sweet chestnut	0.075, 0.15, 0.3	Pigs	E	AA status	Urine MDA	C: ↑ 95.21% (0.075) ↑ 90.49% (0.15%) ↑ 55.65% (0.3%);	NS	17	[63]
			2 control group:			CO: ↓ 31.74% (0.3%)			
			1 lower fat (C),						
			1 oil, rich in seed oil (CO);						
					Plasma MDA	↑ 38.1% (0.075%) ↑ 26.98% (0.3%)	NS		
					UrineiPF2α-VI; TAS, GSH-Px	NS	NS		
				Lymphocyte DNA damage, urinary 8-OHdG excretion and plasma liver enzyme levels	% of DNA in the tail	CO: ↓ 27.5% (0.075%) ↓ 30.83% (0.15%) ↓ 30% (0.3%)	NS		
					OTM	CO: ↓ 53.19% (0.075%) ↓ 55.32% (0.15%) ↓ 53.19% (0.3%)	NS		
					Urine 8-OhdG, GGT, AST	NS	NS		
					ALT	CO: ↓ 33.53% (0.15%) ↓ 31% (0.3%)	NS		
Grape seed polyphenols	0.02, 0.03	Pigs	E, 200 IU/kg	Antioxidant status in serum of sows	TAC, MDA	NS	NS	56	[53]
(GSP)									
					SOD	↑ 64.78% (0.02%) ↑ 76.51% (0.03%)	↑ 46.89% (0.02%) ↑ 57.34% (0.03%)		
					GSH-Px	↑ 34.56% (0.02%) ↑ 48.47% (0.03%)	NS		
Grape seed polyphenols	0.02, 0.03	Pigs	E, 200 IU/kg	Reproductive performance of sows	D of pregnancy, Total born, n. born alive, n. mummies, Total live piglet BIW, Average live piglet BIW, Average weight of weaned piglet, Average weight gain of piglet, n. weaned piglet by sow, Weaning weight by sow	NS	NS	56	[53]
					n. dead fetus	↓ 47.06% (0.03%)	NS		
					Farrowing survival	↑ 9.64% (0.03%)	NS		
					Preweaning survivability	↑ 5.6% (0.02%)	NS		
				Composition in colostrum of sows	Solids-not-fat, Fat, protein, lactose	NS	NS		
					IgM and IgG content	↑ (0.02% = 0.03%) *p* < 0.05	↑ (0.02 = 0.03%) *p* < 0.05		
					colostrum				

Abbreviations: 8-OHdG: 8-hydroxy-2′-deoxyguanosine; AA: antioxidant activity; ACL: antioxidant capacity of lipid-soluble antioxidants; ACW: antioxidant capacity of water-soluble antioxidants; ALT: alanine aminotransferase; AST: aspartate aminotransferase; GGT: gamma-glutamyl transferase; GSH-Px: glutathioneperoxidase; MDA: Malondialdehyde; NS: not significative; OTM: olive tail moment; ROS: reactive oxygen species; TAC: total antioxidant capacity; TAS: total antioxidant status; TBARS: thiobarbituric acid reactive substances; T-SOD: total superoxidedismutase.

**Table 3:** Effects of plant feed additives on feed utilization and growth parameters in ruminants.

PFA	PFA Tested Dose (% in the Diet)	Animal Species Involved	Vitamin/Provitamin Compared	Type of Parameter	Trait Evaluated	Comparison to Negative Control: Effect (PFA Dose)	Comparison to Positive Control: Effect (PFA Dose)	Period of Study (Days)	Reference
Saffron petal extract	0.0025, 0.05	Ovine	E, 225 IU/kg	GROPerf	IBW, FBW, ADG, BWG	NS	NS	56	[70]
				FUPerf	DMI, FCR	NS	NS		
Grape pomace	5, 10	Ovine	E, 500 mg/kg	GROPerf	BBW of lambs, FBW ADG	NS	NS	from birth to 11.5 kg of weight (lambs)	[71]
Yerba Mate (*Ilex paraguariensis)*	3, 3+VitE	Bovine	E, 375 UI/kg DM	FUPerf	DMI	NS	NS	28	[72]
*Andrographis paniculata* (AP), Turmeric acid (TU)	0.5	Goat	E, 400 mg/kg	FUPerf	ADFI	NS	NS	100	[73]
					F:G	↓ 17.33% (TU) ↓ 20.97% (AP)	↓ 14.30% (TU) ↓ 18.08% (AP)		
						↑ 15.17% (AP)	NS		
					FE				
				GROPerf	ADG, IBW, FBW	NS	NS		

Abbreviations: ADG: average daily gain; BBW: birth body weight; BWG: body weight gain; CFI: concentrate feed intake; DMI: dry matter intake; FBW: final body weight; FCR: feed conversion ratio; FE: feed efficiency; F:G feed to gain ratio; FUPerf: feed utilization performances; GROPErf: growth performances; IBW: initial body weight; NS: not significative; PFA: plant feed additive; TFI: total feed intake.

**Table 4:** Effects of plant feed additives on metabolic and hematological parameters and on antioxidant status in ruminants.

	PFA Tested Dose (% in the Diet)	Animal Species Involved	Vitamin/Pro-Vitamin Compared	Type of Parameter	Trait Evaluated	Comparison to Negative Control: Effect (PFA Dose)	Comparison to Positive Control: Effect (PFA Dose)	Period of Study (Days)	Reference
Saffron petal extract	0.0025 (subcutaneous injection, ISPE), 0.05 (oral administration, OSPE)	Ovine	E	Biochemical parameters	Albumin, TP, ALT, AST, Creatinine, Glucose	NS	NS	56	[70]
					TG	NS	↓ 24.57% (ISPE)		
					CHOL (cholesterol)	↓ 21.57% (ISPE)	↑ 35.98% (OSPE)		
				Hematological parameters	WBC, N, E, L, M, RBC, HGB; PCV, MCV, MCHC, Platelets	NS	NS		
				Antioxidant activity (AA) status	GSH-Px	↑ 60.82% (OSPE)	↑ 30.94% (OSPE)		
					SOD	↑ 68.17% (ISPE)	↓ 29.03% (ISPE) ↓ 42.85% (OSPE)		
					MDA, TAC	NS	NS		
				AA in tissues	GSH-Px in muscle, liver, kidney	NS	NS		
					GSH-Px heart	↑ 52.36% (OSPE)	↑ 41.63% (OSPE)		
					SOD muscle, liver, heart	NS	NS		
					SOD kidney	↑ 53.55% (OSPE)	NS		
					MDA in muscle, liver	NS	NS		
					MDA in kidney	↓ 30.77% (ISPE) ↓ 33.54% (OSPE)			
					MDA in heart	NS	↑ 61.29% (ISPE)		
					TAC muscle, liver, kidney, heart	NS	NS		
Plant extract rich in polyphenols (PERP, Rosemary, grape, grapefruit, marigold)	1, along 400 mg/kg of VitE	Bovine	E	Lipid oxidation in tissues	Lipid oxidation	Only the mix of VitE + PERP decreased the lipoperoxidation associated with linseed diets, as shown by a higher lag phase (↑ 47%)	Lower oxidation rate (↓ 48%) compared with group CL.	5 weeks	[80]
			2 control diets: 1 with maize silage (C), 1 with Linseed oil (CL);						
Plant extract rich in polyphenols (PERP) with Vitamin E	1, along 375UI of VitE	Bovine	E, 375 IU/kg	Biochemical parameters	Phospholipids, TG, NEFA (30 d)	NS	NS	30	[81]
			2 control diet: C, CL (linseed oil)						
					Cholesteryl esters (30 d)	C: ↑ 43.51% CL:NS	NS		
					Free CHOL (30 d)	C: ↓ 49.46%	NS		
				Fatty acid (FA) composition of total plasma lipids	Sum of saturated FA	C: ↓ 18.51%	NS		
					Sum of monounsaturated FA	C: ↑ 25.58%	NS		
					Sum of polyunsaturated FA	NS	NS		
					Sum of n-6 PUFA	C: ↓ 31.95%	NS		
					Sum of n-3 PUFA	C: ↑ 344.9%	NS		
					n-6 PUFA/n-3 PUFA	C: ↓ 82%	NS		
					PUFA/SFA	C: ↑ 43.75%	NS		
					Peroxidizability index	C: ↑ 16.3%	NS		
				Liver enzymes	ASAT	C: ↑ 333.63%	NS		
					ALAT, GGT	NS	NS		
				Susceptibility of Plasma to Lipoperoxidation	CD (conjugated dienes) 2 lag phase	C: ↑ 24.14% CL: ↑ 43.05%	↑ 27.06%		
					CD oxidation rate	C: ↓ 35.48% CL: ↓ 48.1%	NS		
					Maximal production of CD	NS	NS		
				Plasma Lipoperoxidation Markers	MDA, TAS	NS	NS		
					α-Tocopherol	C: ↑ 368.85% CL: ↑ 348.28%	↓ 18.75%		
Naringin	0.15, 0.3	Ovine	E, 6000 mg/kg	Plasma lipid peroxidation markers	Total CHOL, HDL CHOL, LDL CHOL (0, 21 d)	NS	NS	21	[79]
					Triacylglycerol (0 d)	NS	NS		
					Triacylglycerol (21 d)	↓ 30.93% (0.015%) ↓ 25.26% (0.03%)	NS		
					Cortisol, TBARS, TAS plasma cortisol and antioxidant parameters on day 21 before the transport period (0 h), immediately after a 4 h transportation period (4 h) and 4 h after	NS	NS		
					having finished the transport (8 h)				
*Andrographis paniculata* (AP), Turmeric acid (TU)	0.5	Goat	E	Plasma lipid oxidation	MDA	↓ 20% (TU) ↓ 25.19% (AP)	NS	100	[73]
					MDA prestarting day	NS	NS		
					MDA 4 weeks	↓ 30.46% (TU) ↓ 41.12% (AP)	NS		
					MDA 8 weeks	NS	NS		
					MDA 14 weeks	↓ 55.03% (AP)	NS		

Abbreviations: ALAT: alanine aminotransferasi; ALT: alanine transaminase; ASAT: aspartate aminotransferase; AST: aspartate aminotransferase; E: eosinophils; FA: fatty acids; GGT: gamma-glutamyl transferase; GSH-Px: glutathione peroxidase; HDL: high-density lipoprotein; HGB: hemoglobin; L: linfocyte; LDL: low density lipoprotein; M: monocyte; MCHC: mean corpuscular hemoglobin concentration; MCV: mean corpuscular volume; MDA: malondialdehyde; N: neutrophils; NEFA: non-esterified fatty acids; PCV: packed cell volume; PUFA: polyunsaturated fatty acid; RBC: red blood cell count; SFA: saturated fatty acids; SOD: superoxide dismutase; TAC: total antioxidant capacity; TAS: total antioxidant status; TBARS: thiobarbituric acid reactive substance; TG: triglycerides; TP: total proteins; WBC: white blood cells.

## Data Availability

The data presented in this study are available free of charge for any user on request from the corresponding authors.
